# Loureirin B Attenuates Methotrexate-Induced Liver Injury Associated with Oxidative Stress, SIRT1 Alterations, and TGF-β/SMAD3-Related Profibrotic Responses

**DOI:** 10.3390/ph19060887

**Published:** 2026-06-02

**Authors:** İrem Hengirmen Acu, Oytun Erbaş

**Affiliations:** 1 Department of Dermatology and Venereology, Faculty of Medicine, Ufuk University, Çankaya, Ankara 06510, Turkey; 2 Faculty of Medicine, BAMER, Biruni University, Istanbul 34015, Turkey; oytunerbas2012@gmail.com

**Keywords:** methotrexate, Loureirin B, hepatotoxicity, oxidative stress, liver fibrosis, SIRT1, TGF-β/SMAD3

## Abstract

**Background:** Methotrexate-induced oxidative stress is mechanistically linked not only to hepatocellular injury but also to DNA damage, indicating that oxidative stress, hepatotoxicity, and genotoxicity represent interconnected manifestations of the same antifolate-driven toxic cascade. Methotrexate (MTX)-induced hepatotoxicity is characterized not only by oxidative stress, but also by progressive fibrotic remodeling driven by activation of the TGF-β/SMAD signaling pathway. **Objective:** We aimed to examine the hepatoprotective effects of Loureirin B, with a particular focus on its anti-fibrotic potential and underlying molecular mechanisms in MTX-induced liver injury. **Methods:** Thirty female Wistar rats were assigned to normal control, MTX, and MTX + Loureirin B groups. Liver injury was induced with a single intraperitoneal MTX dose (20 mg/kg), followed by oral administration of Loureirin B (50 mg/kg/day) for 10 days. Biochemical, molecular, and histopathological analyses were performed, including ALT, AST, ALP, MDA, SIRT1, TGF-β, SMAD3, hydroxyproline, and VEGF levels, alongside the evaluation of necrosis, fibrosis, and inflammatory infiltration. **Results:** MTX induced significant hepatic injury characterized by elevated serum ALT, AST, and ALP levels, increased oxidative stress, suppression of SIRT1, and increased TGF-β and SMAD3 levels, accompanied by elevated collagen-associated markers. Loureirin B treatment significantly reduced the serum liver enzyme levels and oxidative stress, partially restored SIRT1 levels, and decreased fibrosis-associated markers, including hydroxyproline and VEGF. Although the TGF-β levels were significantly reduced following Loureirin B treatment, the reduction in SMAD3 levels did not remain statistically significant after correction for multiple comparisons. Histopathological findings further demonstrated attenuation of fibrosis-associated changes and partial improvement in hepatic architecture. **Conclusions:** Loureirin B may exert protective effects against methotrexate-associated liver injury through the modulation of oxidative stress, partial restoration of SIRT1 levels, attenuation of profibrotic alterations associated with the TGF-β/SMAD pathway, and modulation of VEGF-related responses.

## 1. Introduction

Methotrexate (MTX) is a cornerstone systemic therapy in dermatology, particularly for psoriasis and psoriatic arthritis, due to its well-established efficacy [1]. However, its clinical use is significantly limited by hepatotoxicity, which may range from asymptomatic elevations in liver enzymes to progressive steatosis and fibrosis [2]. This risk is further amplified in patients with metabolic comorbidities such as insulin resistance, obesity, and type 2 diabetes—conditions frequently observed in psoriasis and closely linked to nonalcoholic fatty liver disease (NAFLD) [3,4]. Therefore, despite its therapeutic benefits, concerns regarding liver toxicity necessitate careful patient selection and regular hepatic monitoring during MTX therapy [5].

MTX is a folate analogue containing a pteridine ring linked to p-aminobenzoic acid and glutamate, and its intracellular polyglutamation prolongs hepatic retention and enhances the disruption of one-carbon metabolism, redox balance, and mitochondrial homeostasis in hepatocytes [6,7]. In addition to hepatotoxicity, methotrexate is a documented genotoxic agent, with experimental evidence of DNA strand breaks, micronucleus formation, and multi-organ genomic injury after repeated exposure [8,9,10]. This genotoxic dimension is relevant to the liver because hepatocyte polyploidy may modulate adaptation to oxidative and genotoxic stress during chronic injury and regeneration [11,12,13].

Mechanistically, methotrexate-induced oxidative stress is not merely a parallel event but a central driver of tissue injury, as reactive oxygen species (ROS) amplify lipid peroxidation, inflammatory signaling, and hepatocyte apoptosis while simultaneously facilitating profibrotic responses [6,7,14]. A further layer of complexity is that ROS and transforming growth factor-beta (TGF-β)/SMAD3 signaling form a feed-forward profibrotic loop, since TGF-β increases ROS generation and impairs antioxidant defenses. In contrast, ROS activate latent TGF-β and intensify downstream SMAD-dependent fibrogenic signaling [15,16]. In addition, reduced SIRT1 activity may further amplify oxidative and inflammatory pathways; however, its specific role in MTX-associated hepatic fibrogenesis remains unclear [17,18,19].

Epigenetic mechanisms are central to liver fibrogenesis in general and may also contribute to MTX-associated fibrogenesis, although direct MTX-specific evidence remains limited [20,21]. Within this framework, SIRT1 acts as an NAD+-dependent deacetylase with antifibrotic properties, and reduced SIRT1 activity favors hepatic stellate-cell activation, whereas restoration of SIRT1 can restrain TGF-β/SMAD signaling and fibrogenic transcriptional programs [17,18]. Likewise, suppression of the NFE2L2/NRF2 antioxidant pathway may diminish downstream cytoprotective mediators, such as HMOX1 and NQO1, thereby facilitating ROS accumulation and the persistence of oxidative injury [14,22].

**Figure 1:**
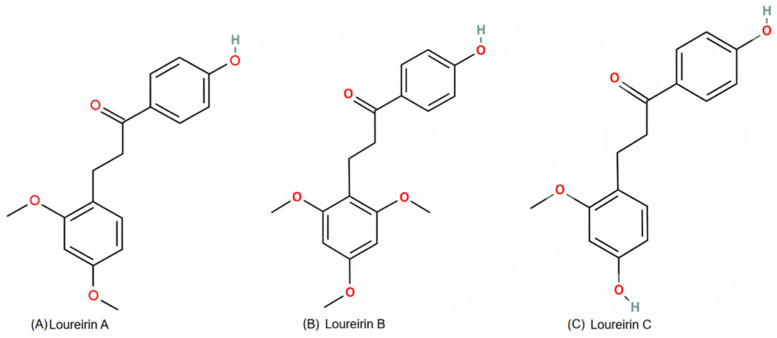
Chemical structures of Loureirin A, Loureirin B, and Loureirin C. Structures were obtained from the PubChem database and redrawn using ChemDraw Professional 20.0 (PerkinElmer Informatics, Waltham, MA, USA).

Loureirins are characteristic phenolic constituents of dragon’s blood derived from Dracaena cochinchinensis, and Loureirins A, B, and C belong to the chalcone/dihydrochalcone branch of flavonoids lato sensu, a structural class whose hydroxylated aromatic rings and carbonyl-containing backbone are compatible with antioxidant and signaling-modulatory activity [23,24]. As a family, dragon’s blood phenolics have been associated with antioxidant, anti-inflammatory, antifibrotic, and tissue-protective activities across several experimental systems, thereby providing a coherent chemical rationale for their potential use against oxidative and fibrogenic liver injury [23,24,25]. Recent work on Loureirin C has further supported the redox-active nature of this family by demonstrating efficient radical-scavenging and intracellular ROS-lowering properties [26] (Figure 1).

Among these bioactive constituents, Loureirin B has attracted particular interest because previous studies have demonstrated its antifibrotic potential through modulation of TGF-β/Smad signaling and suppression of extracellular matrix accumulation. In addition, Loureirin B has been reported to inhibit hepatic stellate-cell activation via the miR-148-3p/Wnt/β-catenin pathway and to ameliorate cholestatic liver fibrosis through mechanisms associated with AKT/mTOR/ATG7 signaling [27,28,29]. Experimental studies have also demonstrated that Loureirin B inhibits profibrotic signaling in fibroblast models and suppresses hepatic stellate-cell activation in cholestatic liver injury models [27,29]. In vivo efficacy has been reported with oral doses up to 50 mg/kg/day in rats, supporting the biological plausibility of the present dose while still requiring model-specific justification [30].

In light of these findings, Loureirin B may represent a promising candidate for mitigating MTX-induced hepatic injury and fibrotic responses. Therefore, the present study aimed to determine whether Loureirin B attenuates methotrexate-induced liver injury and fibrogenic remodeling in rats by reducing oxidative stress, restoring SIRT1-associated cytoprotection, and modulating TGF-β/SMAD3-related profibrotic signaling.

## 2. Results

### 2.1. Histopathological Findings

**Figure 2:**
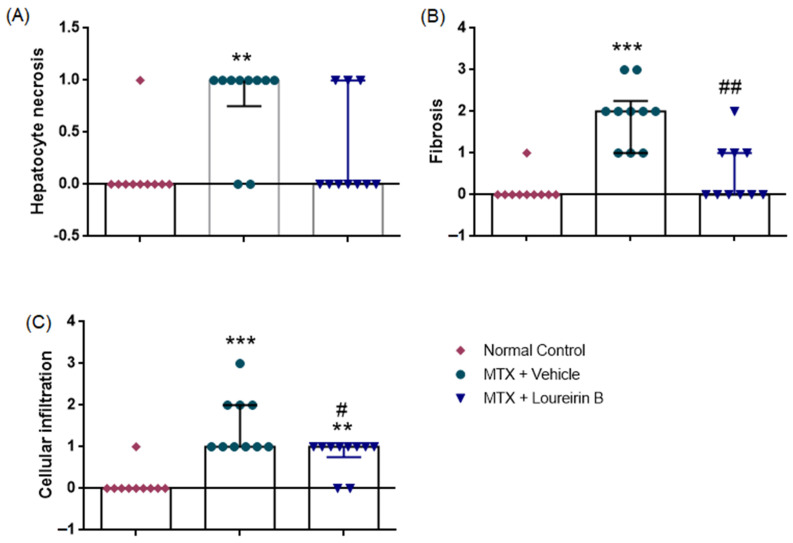
Effects of methotrexate (MTX) and Loureirin B on histopathological injury scores: (**A**) hepatocyte necrosis, (**B**) fibrosis, and (**C**) cellular infiltration. Data are presented as median and interquartile range [IQR]. Statistical analyses were performed using the Kruskal–Wallis test followed by pairwise Mann–Whitney U tests with Bonferroni correction. ** *p* < 0.01, *** *p* < 0.001 vs. normal control group; # *p* < 0.017, ## *p* < 0.01 vs. MTX group.

As presented in Figure 2 and Table 1, histopathological injury scores differed significantly among the experimental groups for necrosis (Kruskal–Wallis χ^2^(2) = 10.472, *p* = 0.005), fibrosis (χ^2^(2) = 18.908, *p* < 0.001), and cellular infiltration (χ^2^(2) = 18.964, *p* < 0.001). Necrosis scores were significantly higher in the MTX group (median [IQR]: 1 [0.75–1]) compared to the normal control group (0 [0–0]) (Mann–Whitney U = 15.0, *p* = 0.002). Necrosis scores tended to be lower in the MTX + Loureirin B group compared with the MTX group; however, this difference did not remain statistically significant after Bonferroni correction (adjusted significance threshold: *p* < 0.017) (U = 25.0, *p* = 0.028). Fibrosis scores were markedly increased in the MTX group (2 [1–2.25]) compared with the normal control group (0 [0–0]) (U = 1.5, *p* < 0.001). Loureirin B treatment significantly reduced the fibrosis scores (1 [0–1]) compared to MTX (U = 10.0, *p* = 0.002). Similarly, cellular infiltration scores were elevated in the MTX group (1 [1–2]) compared to the normal control group (0 [0–0]) (U = 3.0, *p* < 0.001). Loureirin B treatment reduced the cellular infiltration scores (1 [0.75–1]) compared to MTX (U = 24.0, *p* = 0.015). There was no significant difference between the normal control and MTX + Loureirin B groups in terms of necrosis (U = 40.0, *p* = 0.276) and fibrosis scores (U = 34.5, *p* = 0.121). In contrast, cellular infiltration scores were significantly higher in the MTX + Loureirin B group compared to the normal control group (U = 15.0, *p* = 0.002).

#### Histopathological Findings in Figure 3

Histopathological examination of liver sections revealed well-preserved hepatic architecture in the normal control group, characterized by normal hepatocyte morphology, intact central veins, and regular portal areas. The MTX group showed marked hepatic damage, including necrosis, periportal fibrosis, inflammatory infiltration, and disruption of normal architecture. In contrast, the MTX + Loureirin B group demonstrated reduced tissue injury, with milder necrosis and inflammation, indicating partial preservation of liver structure despite residual changes.

**Figure 3:**
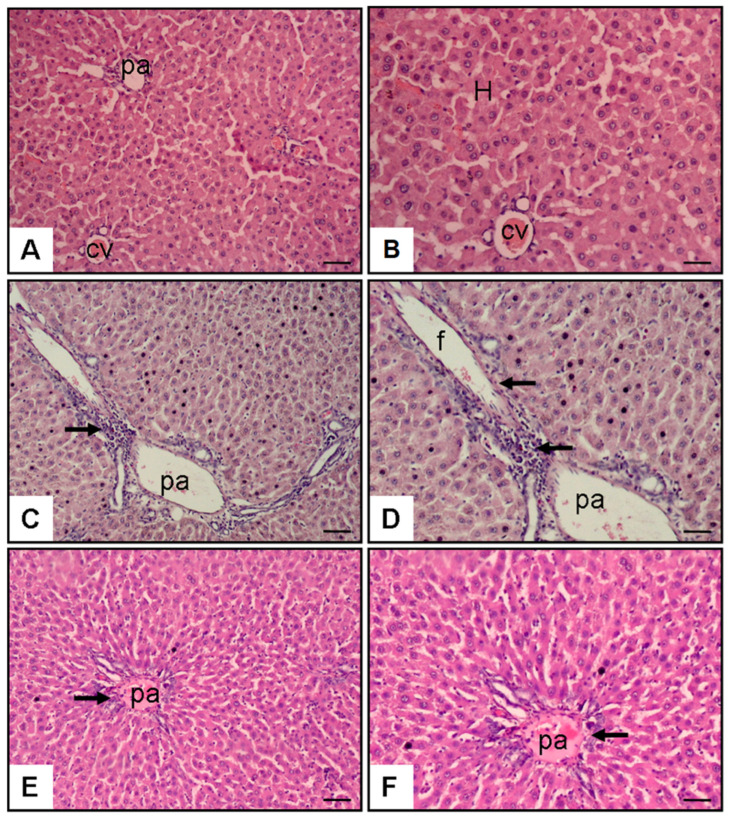
Representative H&E-stained liver sections (×20–×40). The normal control group (**A**,**B**) showed normal hepatic architecture. The MTX group (**C**,**D**) demonstrated fibrosis-associated alteration and inflammatory infiltration, particularly in the portal area. In contrast, the MTX + Loureirin B group (**E**,**F**) showed attenuation of these alterations with partial restoration of liver architecture. Abbreviations: H, hepatocytes; cv, central vein; pa, portal area; f, fibrosis. Scale bar: ×20 and ×40 magnifications.

### 2.2. Biochemical Findings

#### Serum Liver Enzyme Levels (ALT, AST, and ALP)

**Figure 4:**
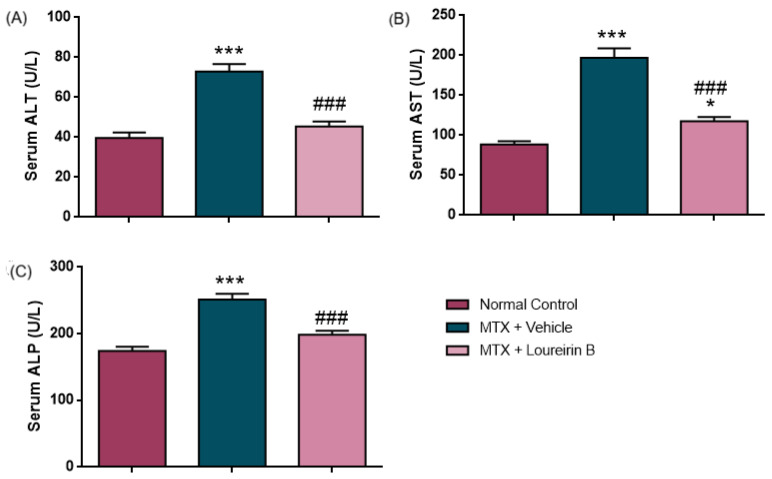
Effects of MTX and Loureirin B on the serum liver enzyme levels. (**A**) ALT, (**B**) AST, and (**C**) ALP levels. MTX increased the ALT, AST, and ALP levels, whereas Loureirin B attenuated these changes. Data are mean ± SEM (*n* = 10 per group). * *p* < 0.05, *** *p* < 0.001 vs. normal control group; ### *p* < 0.001 vs. MTX group.

As presented in Figure 4 and Table 2, serum ALT, AST, and ALP levels differed significantly among the experimental groups. Serum ALT levels were significantly higher in the MTX group (72.88 ± 3.75 IU/L) than in the normal control group (39.63 ± 2.71 IU/L; F(2,27) = 34.83, *p* < 0.0001). Following Loureirin B treatment, ALT levels decreased to 45.44 ± 2.40 IU/L (*p* < 0.0001 vs. MTX) and did not significantly differ from those of the normal control group (*p* = 0.3728). Similarly, the serum AST levels showed significant intergroup differences (F(2,27) = 51.05, *p* < 0.0001), with markedly elevated values in the MTX group (197.0 ± 11.89 IU/L) compared with the normal control group (88.50 ± 4.05 IU/L, *p* < 0.0001). Loureirin B treatment reduced the AST levels to 117.6 ± 5.24 IU/L (*p* < 0.0001 vs. MTX), although the values remained slightly higher than those of the normal control group (*p* = 0.0370). Serum ALP levels also differed significantly among groups (F(2,27) = 30.74, *p* < 0.0001). ALP levels were significantly increased in the MTX group (251.3 ± 8.64 IU/L) compared with the normal control group (174.0 ± 6.28 IU/L, *p* < 0.0001). Loureirin B treatment reduced the ALP levels to 198.3 ± 6.20 IU/L (*p* < 0.0001 vs. MTX), although this reduction did not reach statistical significance compared with the normal control group (*p* = 0.0580).

### 2.3. Hepatic Oxidative Stress and SIRT1 Levels

**Figure 5:**
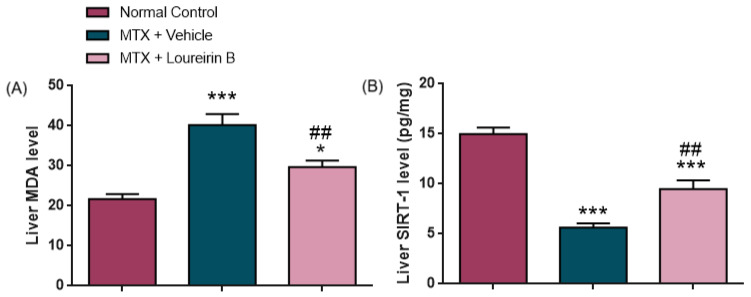
Effects of MTX and Loureirin B on hepatic oxidative stress markers. (**A**) Liver MDA and (**B**) liver SIRT1 levels. MTX increased hepatic MDA levels and reduced hepatic SIRT1 levels, whereas Loureirin B attenuated these alterations. Data are presented as mean ± SEM (n = 10 per group). * *p* < 0.05, *** *p* < 0.001 vs. normal control group; ## *p* < 0.01 vs. MTX group.

Liver MDA levels differed significantly among groups (F(2,27) = 22.64, *p* < 0.0001), with higher values in the MTX group (40.23 ± 2.70 nmol/g tissue) compared with the normal control group (21.68 ± 1.29 nmol/g tissue, *p* < 0.0001). Loureirin B treatment reduced the MDA levels (29.74 ± 1.57 nmol/g tissue; *p* = 0.0021 vs. MTX), although the values remained higher than those of the normal control group (*p* = 0.0187). Similarly, the liver SIRT1 levels showed a significant group effect (F(2,27) = 47.55, *p* < 0.0001). SIRT1 levels were significantly lower in the MTX group (5.63 ± 0.43 pg/mg) than in the normal control group (14.99 ± 0.66 pg/mg, *p* < 0.0001). Following Loureirin B treatment, SIRT1 levels increased to 9.47 ± 0.88 pg/mg (*p* = 0.0013 vs. MTX), but remained below normal control values (*p* < 0.0001) (Figure 5, Table 2).

### 2.4. Hepatic TGF-β/SMAD3-Related Profibrotic Markers and VEGF Levels

**Figure 6:**
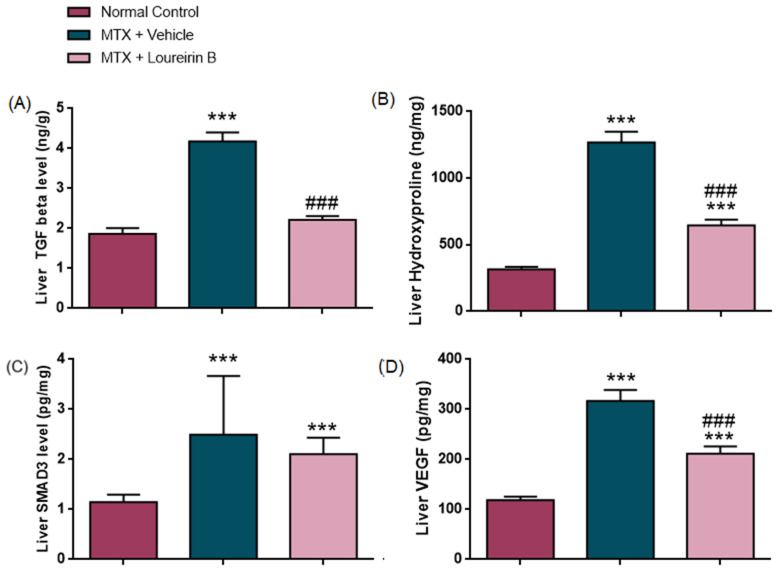
Effects of MTX and Loureirin B on profibrotic and angiogenic markers. (**A**) Liver TGF-β, (**B**) liver hydroxyproline, (**C**) liver SMAD3, and (**D**) liver VEGF levels. MTX increased all parameters, whereas Loureirin B partially attenuated these alterations. Data are presented as mean ± SEM for TGF-β, hydroxyproline, and VEGF, and as median [IQR] for SMAD3 (*n* = 10 per group). Statistical analyses for SMAD3 were performed using the Kruskal–Wallis test followed by pairwise Mann–Whitney U tests with Bonferroni correction. *** *p* < 0.001 vs. normal control group; ### *p* < 0.001 vs. MTX group.

As presented in Table 2 and Figure 6, all parameters showed significant differences among groups (TGF-β: F(2,27) = 58.02; hydroxyproline: F(2,27) = 80.75; SMAD3: χ^2^(2) = 21.47; VEGF: F(2,27) = 39.53; all *p* < 0.001).

Liver TGF-β levels were higher in the MTX group (4.18 ± 0.22 ng/g) than in the normal control group (1.86 ± 0.15 ng/g, *p* < 0.0001). After Loureirin B treatment, TGF-β decreased to 2.21 ± 0.10 ng/g (*p* < 0.0001 vs. MTX) and was comparable to the normal control group (*p* = 0.3019). Hydroxyproline levels were elevated in the MTX group (1268.45 ± 80.85 ng/mg) compared to the normal control (315.80 ± 18.61 ng/mg, *p* < 0.0001). With treatment, levels decreased to 646.21 ± 42.55 ng/mg (*p* < 0.0001 vs. MTX), although they remained above normal control values (*p* = 0.0005).

SMAD3 levels were significantly higher in the MTX group (2.50 [2.21–3.66] pg/mg) compared with the normal control group (1.15 [0.93–1.30] pg/mg, *p* < 0.001). Following Loureirin B treatment, the SMAD3 levels decreased to 2.10 [1.86–2.43] pg/mg; however, this reduction did not remain statistically significant after Bonferroni correction for multiple comparisons (adjusted significance threshold: *p* < 0.017; *p* = 0.029 vs. MTX). SMAD3 levels in the MTX + Loureirin B group remained significantly higher than those in the normal control group (*p* < 0.001). VEGF levels were also increased in the MTX group (316.41 ± 22.21 pg/mg) compared to the normal control (118.90 ± 7.04 pg/mg, *p* < 0.0001). Following treatment, VEGF decreased to 211.97 ± 14.08 pg/mg (*p* = 0.0002 vs. MTX), yet remained higher than the normal control values (*p* = 0.0008).

### 2.5. Body Weight Analysis

**Figure 7:**
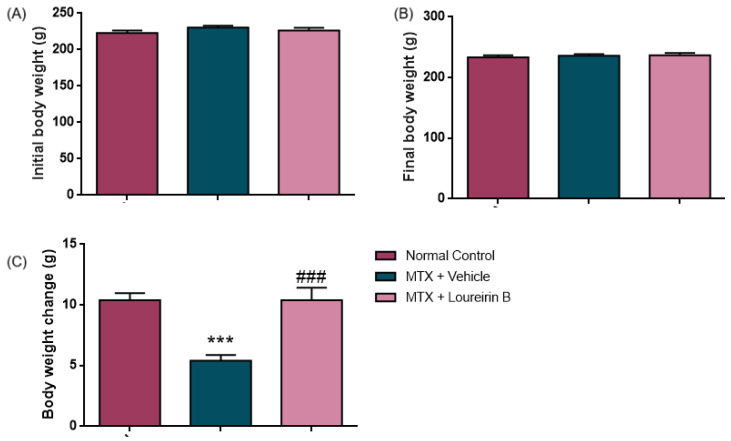
Effects of MTX and Loureirin B treatment on body weight parameters. (**A**) Initial body weight, (**B**) final body weight, and (**C**) body weight change during the experimental period. No significant differences were observed among groups in the initial or final body weights (*p* > 0.05). Body weight gain was significantly reduced in the MTX + Vehicle group compared with the Normal Control group, whereas Loureirin B treatment attenuated this reduction. Data are presented as mean ± SEM (n = 10 per group). *** *p* < 0.001 vs. Normal Control group; ### *p* < 0.001 vs. MTX + Vehicle group.

Body weight data were normally distributed according to the Shapiro–Wilk test (all *p* > 0.05); therefore, parametric analyses were performed, and data are presented as mean ± SEM. Initial body weights were 222.9 ± 3.5 g in the Normal Control group, 230.5 ± 2.5 g in the MTX + Vehicle group, and 226.4 ± 3.7 g in the MTX + Loureirin B group, with no significant difference among groups (*p* = 0.278). Final body weights were 233.3 ± 3.6 g, 235.9 ± 2.7 g, and 236.8 ± 3.8 g, respectively, and also did not differ significantly among groups (*p* = 0.753). However, body weight change differed significantly among groups (one-way ANOVA, F(2,27) = 15.71, *p* < 0.0001). Tukey post hoc analysis revealed significantly lower body weight gain in the MTX + Vehicle group compared with the Normal Control group (*p* = 0.0001). In contrast, the MTX + Loureirin B group showed weight gain comparable to that of the controls (*p* > 0.9999). In addition, body weight gain was significantly higher in the MTX + Loureirin B group than in the MTX + Vehicle group (*p* = 0.0001), suggesting that Loureirin B attenuated MTX-associated impairment in body weight gain (Figure 7).

## 3. Discussion

In this study, MTX induced marked hepatotoxicity, reflected by increased serum ALT, AST, and ALP levels, elevated hepatic MDA levels, reduced SIRT1 levels, elevated TGF-β and SMAD3 levels accompanied by profibrotic changes, increased hydroxyproline and VEGF levels, and histological evidence of necrosis, fibrosis, and inflammation. Loureirin B treatment reduced the serum liver enzyme and oxidative stress marker levels, partially restored SIRT1 levels, and attenuated fibrosis-associated alterations. Histopathological evaluation also demonstrated reduced fibrosis and inflammatory changes, together with partial improvement in necrotic alterations.

The histopathological changes observed—hepatocellular necrosis, inflammatory infiltration, and fibrotic remodeling—are consistent with experimental MTX-induced hepatotoxicity, in which structural liver injury is accompanied by biochemical evidence of hepatocellular damage [6,19,31]. Serum ALT and AST are well-established markers of hepatocellular injury; ALT is predominantly cytosolic and relatively liver-specific, whereas AST is present in both cytosolic and mitochondrial fractions and is therefore less specific [32,33]. In contrast, ALP is enriched at the canalicular membrane, and its elevation is more suggestive of accompanying hepatobiliary/canalicular involvement and a possible mixed injury pattern than that of simple hepatocyte enzyme leakage [34]. In the present study, the marked increases in serum ALT, AST, and ALP after MTX administration, together with evident necrosis, support a close association between tissue injury and biochemical disruption.

Previous studies indicate that oxidative stress and inflammatory signaling are major contributors to MTX-induced liver injury [6,7]. In this context, the increase in hepatic MDA observed in the present study supports the hypothesis of enhanced lipid peroxidation and oxidative membrane injury. However, MDA should be interpreted as an indirect marker of oxidative stress rather than as a stand-alone mechanistic readout [35,36]. Such membrane damage can impair cellular integrity and facilitate the release of ALT and AST into the circulation even before complete cell death. In comparison, the rise in ALP may reflect concurrent dysfunction at the hepatobiliary/canalicular interface accompanying parenchymal injury rather than simple cytosolic enzyme leakage [32,34]. Loureirin B treatment reduced the serum ALT, AST, and ALP levels, hepatic MDA levels, and was accompanied by lower necrosis scores, suggesting the preservation of hepatocellular and canalicular membrane integrity through the attenuation of oxidative injury.

Beyond hepatotoxicity, methotrexate has also been associated with genotoxic effects in both experimental and clinical studies, including DNA strand breaks, micronucleus formation, and chromosomal injury [8,9,10]. Although dedicated genotoxicity assays were not included in the present study, oxidative DNA damage may coexist with the biochemical and histopathological evidence of hepatocellular stress observed after MTX administration [6,9]. From this perspective, the reduction in oxidative stress markers observed following Loureirin B treatment may also indirectly suggest the attenuation of oxidative conditions that contribute to MTX-associated genomic injury. However, further studies incorporating direct genotoxicity endpoints are required to clarify this relationship.

MTX administration showed a clear increase in hepatic TGF-β and SMAD3 levels, consistent with the activation of a profibrotic TGF-β/SMAD3-related response in which TGF-β signaling has been associated with SMAD-dependent stellate cell activation and matrix deposition [16,37]. Previous studies have also demonstrated that this pathway is closely linked to oxidative stress, as reactive oxygen species can activate TGF-β. At the same time, TGF-β further enhances ROS production, creating a self-amplifying cycle [6,15]. In line with earlier MTX models [31,38], our findings are consistent with the involvement of this axis in MTX-associated profibrotic changes. Following Loureirin B treatment, the hepatic TGF-β levels were significantly reduced, whereas the SMAD3 levels showed a numerical decline that did not remain statistically significant after correction for multiple comparisons. These findings may suggest partial attenuation of this profibrotic signaling response.

Hydroxyproline analysis further demonstrated increased collagen-associated matrix accumulation following MTX administration, consistent with its established use as a marker of collagen deposition and profibrotic remodeling [39,40,41]. This increase paralleled the histopathological fibrosis scores, supporting enhanced extracellular matrix deposition in the MTX-treated liver tissue. Similar findings have been reported in experimental models, where hydroxyproline levels were associated with the severity of profibrotic changes [42]. Loureirin B treatment reduced the hydroxyproline levels, suggesting decreased collagen accumulation and the attenuation of profibrotic remodeling.

MTX treatment in this study was associated with increased VEGF levels, which may reflect angiogenesis-related and profibrotic responses linked to hypoxia-associated signaling and endothelial–stellate cell interactions [43,44]. Elevated VEGF levels have been associated with both vascular remodeling and progression of profibrotic changes [45,46], although VEGF may also contribute to tissue repair depending on the biological context [47]. In the present study, the reduction in VEGF levels following Loureirin B treatment may indicate the attenuation of this angiogenesis-related response; however, given VEGF’s dual role in both fibrosis and tissue repair, these findings should be interpreted cautiously.

SIRT1 is known as a key regulator of cellular stress responses, with protective roles in redox balance and inflammation. In MTX-induced hepatotoxicity, previous studies have shown that SIRT1 is downregulated, which is associated with increased oxidative stress and inflammatory injury [48]. In the present study, a similar decrease in hepatic SIRT1 levels was observed following MTX administration.

Beyond oxidative stress, SIRT1 has also been implicated in fibrogenic processes by limiting hepatic stellate cell activation and reducing profibrotic gene expression [17]. Previous studies have further suggested potential interactions between SIRT1 and TGF-β/SMAD-related signaling pathways in the regulation of profibrotic responses [18,30]. In the present study, the increase in SIRT1 levels observed following Loureirin B treatment may suggest the partial restoration of cytoprotective mechanisms associated with the attenuation of oxidative stress and profibrotic processes.

Loureirin B, a dihydrochalcone-type flavonoid derived from Resina Draconis, is known for its antioxidant and anti-inflammatory properties and has been proposed as a potential modulator of MTX-induced liver injury [23]. Previous studies have reported that Loureirin B exhibits antifibrotic properties through multiple mechanisms. In hepatic models, it has been shown to inhibit hepatic stellate cell (HSC) proliferation and promote apoptosis via Wnt/β-catenin- and miR-148-3p-related pathways [28]. In addition, in cholestatic fibrosis models, Loureirin B has been associated with the enhancement of HSC autophagy through AKT/mTOR/ATG7-related signaling [29]. Other experimental studies have also suggested that Loureirin B may modulate profibrotic signaling responses, including TGF-β/SMAD-related pathways [27,49,50].

Our findings should also be interpreted in the context of a broader literature showing that MTX-induced oxidative liver damage can be mitigated by several natural or repurposed compounds. For example, 18β-glycyrrhetinic acid, glabridin, punicalagin, paeonol, lutein, and fisetin have all been reported to reduce biochemical injury, oxidative stress, inflammation, or profibrotic alterations in experimental methotrexate hepatotoxicity models, frequently with the restoration of antioxidant defenses and improvement in tissue architecture [14,19,48,51,52,53]. This convergence suggests that the protective effect observed for Loureirin B fits a recurring mechanistic pattern in which redox regulation and the attenuation of profibrotic signaling are closely interconnected rather than independent processes [15,16].

In the present study, these mechanisms appear to be reflected at multiple levels. Loureirin B treatment was associated with increased SIRT1 levels and reduced lipid peroxidation, which may reflect the attenuation of oxidative conditions associated with profibrotic responses and extracellular matrix accumulation. The concurrent reduction in VEGF levels may indicate the modulation of angiogenesis-related responses associated with profibrotic remodeling [15,37,43]. However, the hydroxyproline levels remained elevated compared with the normal control group, and the SMAD3 levels did not remain significantly reduced after correction for multiple comparisons, suggesting that extracellular matrix remodeling and profibrotic signaling may persist despite partial biochemical and histopathological improvement. This observation may also be influenced by the context-dependent role of VEGF, which can contribute to both fibrogenesis and tissue repair [47].

An additional interpretive aspect of the present findings is the potential involvement of epigenetic signaling in MTX-induced profibrotic responses. Hepatic fibrogenesis is increasingly recognized to involve not only cytokine-mediated pathways but also epigenetic regulation of the stellate-cell phenotype, including histone modification and non-coding RNA-associated mechanisms [20,21]. Within this framework, reduced SIRT1 activity has been associated with enhanced stellate-cell activation and TGF-β/SMAD-related profibrotic signaling, whereas the preservation of SIRT1 may contribute to the attenuation of oxidative stress and profibrotic responses [17,18]. In addition, impairment of the NFE2L2 antioxidant axis may facilitate ROS accumulation and persistence of oxidative injury, thereby contributing to profibrotic remodeling [14]. From this perspective, the findings observed following Loureirin B treatment may be interpreted within a broader epigenetic–redox framework potentially associated with MTX-induced profibrotic alterations [23,24,25,29].

In the present study, MTX did not significantly alter the absolute final body weight, but it clearly reduced the expected body weight gain, suggesting subacute systemic toxicity rather than overt cachexia. This interpretation is consistent with MTX’s established ability to induce oxidative stress, inflammatory injury, and metabolic disturbances during hepatotoxicity, which may indirectly impair normal weight gain over short experimental periods [6,53]. Notably, Loureirin B restored body weight gain to a level comparable with the normal control group without increasing body weight beyond the control values, suggesting partial preservation of systemic physiological status during MTX challenge. Previous experimental toxicology studies have also demonstrated that female rats may exhibit altered body weight gain and increased susceptibility to hepatotoxic injury following exposure to certain hepatotoxic compounds, potentially related to sex-dependent differences in hepatic xenobiotic metabolism and oxidative stress responses [54]. Because food intake was not measured in the present study, these findings should be interpreted as supportive rather than definitive evidence of improved systemic well-being.

Only female rats were used in the present study to maintain biological sex homogeneity within the experimental design and to minimize the potential confounding effects of sex-related variability in hepatic drug metabolism and toxicity responses. Previous experimental toxicology studies have demonstrated sex-dependent differences in hepatic xenobiotic metabolism and susceptibility to hepatotoxic injury [54,55]. In addition, the use of female Wistar/Wistar-albino rats in previous studies investigating MTX-induced hepatotoxicity provided methodological precedence for the present experimental model [56,57,58].

## 4. Materials and Methods

### 4.1. Animals

A total of 30 female Wistar albino rats, aged 10–12 weeks and weighing 210–240 g, were enrolled in the present experimental study. Animals were housed under standard laboratory conditions and maintained with unrestricted access to tap water and a standard laboratory rodent diet (RM3 (P), SDS^®^, Essex, UK) throughout the experimental period. Before the initiation of experimental procedures, all animals underwent an acclimatization period at the Experimental Animal Research Center of Science University, from which they were obtained. All experimental protocols and animal handling procedures were performed in accordance with the ARRIVE (Animal Research: Reporting of In Vivo Experiments) guidelines and the Guide for the Care and Use of Laboratory Animals issued by the National Institutes of Health (USA). Ethical approval was granted by the Institutional Animal Ethics Committee of Science University (Approval No: 419033; Approval Date: 20 March 2023).

Animals were excluded from the study if any of the following predefined humane endpoint criteria were observed: more than 15% loss of baseline body weight, marked reduction in food or water intake, severe impairment of mobility or inability to ambulate normally, markedly reduced response to external stimuli, severe lethargy, deterioration in general condition, signs of uncontrolled pain, development of infection, severe wound complications, unexpected neurological abnormalities, or recommendation for humane euthanasia by the attending veterinarian.

### 4.2. Drugs and Chemicals

Loureirin B (PHL84235, purity ≥ 98% by HPLC) was purchased from Sigma-Aldrich (St. Louis, MO, USA). Methotrexate (METHOTREXATE-KOÇAK^®^, 50 mg/5 mL injectable solution; Koçak Farma, Istanbul, Türkiye) was used in this study. Anesthesia was induced with ketamine hydrochloride (Ketasol^®^, Richter Pharma AG, Wels, Austria) and xylazine hydrochloride (Rompun^®^, Bayer, Leverkusen, Germany). Loureirin B was prepared as a freshly mixed, homogeneous suspension in a vehicle containing 5% DMSO and 95% distilled water, and vortexed immediately before administration. The suspension was administered by oral gavage at a dose of 50 mg/kg in a final volume of 1 mL/kg.

No separate preliminary pilot study was performed before the main experiment. Dose selection and treatment duration were determined based on previously published studies and the biological rationale of the MTX-induced liver injury model. The 50 mg/kg/day oral dose of Loureirin B was selected according to previous experimental studies, demonstrating biological efficacy and tolerability at similar dose ranges in inflammatory, metabolic, and hepatic injury models, including studies using 40–50 mg/kg regimens and oral gavage administration in rats [29,30,59]. The 10-day treatment period was selected to evaluate the acute-to-subacute phase of MTX-induced hepatic and profibrotic alterations in a single-dose MTX model, consistent with previously reported experimental protocols [31,60].

### 4.3. Experimental Protocol

Thirty female Wistar albino rats were enrolled in the experimental protocol. Animals were allocated to study groups using a computerized randomization procedure to minimize selection bias. To establish the hepatic injury model, twenty animals received a single intraperitoneal injection of methotrexate (MTX) at 20 mg/kg. The remaining animals served as the healthy control group and were not treated with MTX.

Group 1 (Normal control; *n* = 10): Rats received the corresponding vehicle solution (5% DMSO in distilled water, 1 mL/kg/day, oral gavage) once daily for 10 consecutive days.

Group 2 (Toxic control/negative control; MTX + Vehicle, *n* = 10): Rats received a single intraperitoneal dose of methotrexate (20 mg/kg). Thirty minutes after MTX administration, the animals received the vehicle solution (5% DMSO in distilled water, 1 mL/kg/day, oral gavage) once daily for 10 consecutive days.

Group 3 (MTX + Loureirin B, *n* = 10): Rats received a single intraperitoneal dose of methotrexate (20 mg/kg). Thirty minutes after MTX administration, Loureirin B treatment (50 mg/kg/day, oral gavage) was initiated and continued once daily for 10 consecutive days.

No animal mortality occurred during the experimental period, and all animals survived until the planned termination of the study on day 10.

**Figure 8:**
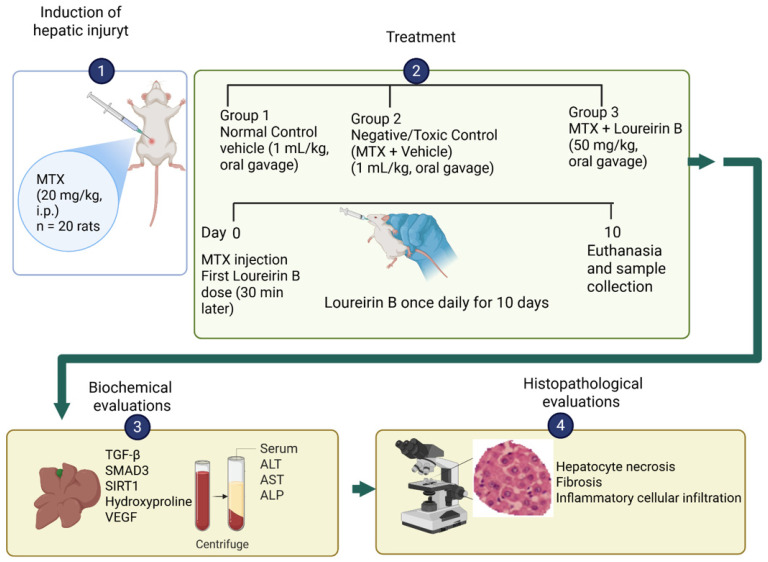
Experimental design and treatment timeline of the study. A single intraperitoneal methotrexate (MTX) injection (20 mg/kg) was administered on Day 0 to induce hepatic injury. Loureirin B treatment (50 mg/kg, oral gavage) was initiated 30 min after MTX administration and continued once daily for 10 consecutive days. Animals were euthanized, and tissue samples were collected on Day 10. Group 1 served as the normal control group and received vehicle only. In contrast, Group 2 received MTX + vehicle, and Group 3 received MTX + Loureirin B. Biochemical evaluations included serum ALT, AST, and ALP, as well as liver TGF-β, SMAD3, SIRT1, hydroxyproline, and VEGF levels. Histopathological assessments included hepatocyte necrosis, fibrosis, and cellular infiltration. Abbreviations: MTX, methotrexate; i.p., intraperitoneal.

After the experimental period, animals were deeply anesthetized with ketamine (75 mg/kg) and xylazine (15 mg/kg). Following confirmation of an adequate anesthetic depth, blood samples were obtained via cardiac puncture. Subsequently, euthanasia was performed by cervical dislocation while animals remained under deep anesthesia. Liver specimens were then carefully harvested and processed for subsequent histopathological and biochemical analyses (Figure 8).

### 4.4. Histopathological Evaluation

#### Tissue Processing, Microscopy, and Histopathological Scoring

For histopathological evaluation, liver specimens were preserved in 10% neutral-buffered formalin and subsequently processed routinely, followed by paraffin embedding. Paraffin blocks were sectioned at a thickness of 4 μm using a rotary microtome. Tissue sections were then stained with Harris Modified Hematoxylin (Cat. No. HHS32; Sigma-Aldrich, St. Louis, MO, USA) and aqueous Eosin Y (Cat. No. HT110232; Sigma-Aldrich, St. Louis, MO, USA) to assess histomorphological alterations. Microscopic examinations were performed using an Olympus BX51 light microscope (Olympus Corp., Tokyo, Japan), and representative photomicrographs were acquired with an Olympus C-5050 digital imaging system (Olympus Corp., Tokyo, Japan).

Histopathological evaluation was performed independently by two blinded investigators to minimize observational bias. To reduce sampling bias, five non-overlapping high-power fields (HPFs; 400× magnification) per animal were systematically evaluated from different regions of each liver section, including periportal and parenchymal areas. One standardized liver section from each animal was used for histopathological scoring. Liver injury was assessed semi-quantitatively based on hepatocyte necrosis, fibrosis, and inflammatory infiltration [15], each graded on a 0–4 scale (0: absent; 4: >50%). Total histopathological scores were calculated by summing individual parameters. Interobserver agreement was quantified using weighted Cohen’s κ statistics (quadratic weights), demonstrating strong agreement between observers (κ = 0.85, 95% CI: 0.78–0.91; *p* < 0.001).

### 4.5. Liver Biochemical Analysis

Following tissue collection, liver samples were immediately harvested, washed with ice-cold physiological saline to remove residual blood, and preserved at −80 °C until further processing. For biochemical assessments, tissue specimens were homogenized in chilled phosphate-buffered saline (PBS; pH 7.4) at a 1:5 (*w*/*v*) ratio, then centrifuged at 5000× *g* for 15 min. The resulting supernatants were separated and used for subsequent analyses. Total protein concentrations were quantified according to the Bradford protein assay and used for data normalization [61]. To reduce potential assessment bias, all biochemical analyses were performed by investigators blinded to the experimental groups. Reproducibility between independent measurements was assessed using the intraclass correlation coefficient (ICC) with a two-way mixed-effects model and absolute agreement approach, revealing a high degree of reliability (ICC = 0.93, 95% CI: 0.88–0.96; *p* < 0.001). Enzyme-linked immunosorbent assay measurements were performed in duplicate according to the manufacturer’s recommended protocols to ensure analytical consistency. Optical density values were measured with a microplate spectrophotometer (Multiskan GO, Thermo Fisher Scientific, Waltham, MA, USA), and sample concentrations were determined from standard calibration curves.

Liver transforming growth factor-beta (TGF-β), sirtuin-1 (SIRT1), hydroxyproline (Hyp), vascular endothelial growth factor (VEGF), and SMAD3 levels were measured in rat liver tissue homogenates using rat-specific ELISA kits obtained from MyBioSource (San Diego, CA, USA), CUSABIO (Wuhan, China), and ELK Biotechnology (Wuhan, China). The following kits were used: TGF-β (Cat. No. MBS160117), SIRT1 (Cat. No. ELK6192), hydroxyproline/Hyp (Cat. No. CSB-E08838r), VEGF (Cat. No. MBS724516), and SMAD3 (Cat. No. ELK0025), according to the manufacturers’ instructions.

Lipid peroxidation was assessed by measuring malondialdehyde (MDA) levels using the thiobarbituric acid reactive substances (TBARS) assay, as previously described by Draper and Hadley with minor modifications [62]. Liver homogenates were mixed with trichloroacetic acid (TCA) and thiobarbituric acid (TBA) reagent and incubated at 95–100 °C for 60 min. After cooling and centrifugation, absorbance was measured spectrophotometrically at 535 nm using a microplate reader. MDA concentrations were expressed as nmol/g protein. The TBARS method remains a widely accepted approach for assessing oxidative lipid peroxidation in biological tissues [35].

#### Determination of Serum AST, ALP, and ALT Levels

For serum preparation, blood specimens obtained by cardiac puncture were transferred into serum-separator collection tubes and maintained at room temperature for approximately 30 min to allow complete coagulation. Following clot formation, samples were centrifuged at 1500× *g* for 10 min, and the resulting serum fraction was isolated and preserved at −80 °C until biochemical evaluation. Serum activities of ALT, AST, and ALP were quantified by kinetic assay methods using commercially available reagent kits and analyzed with an automated chemistry analyzer (Beckman Coulter LX-2000; Beckman Coulter, Brea, CA, USA) following the manufacturer’s recommended protocols.

### 4.6. Statistical Analysis

Statistical processing of the study data was conducted using SPSS software (version 19.0; IBM Corp., Armonk, NY, USA). Before comparative analyses, the assumption of normality was evaluated through the Shapiro–Wilk test. Variables exhibiting Gaussian distribution patterns were assessed by one-way analysis of variance, and intergroup differences were subsequently explored using Tukey’s post hoc procedure. Data that did not satisfy parametric assumptions, including histopathological scores and SMAD3 measurements, were analyzed using nonparametric methods. Specifically, overall group differences were assessed using the Kruskal–Wallis test, followed by Mann–Whitney U testing for pairwise comparisons when appropriate. To control for type I error associated with multiple testing, the Bonferroni correction was applied, and the adjusted threshold for statistical significance was defined as *p* < 0.017. Continuous variables with normal distribution are reported as the mean ± standard error of the mean (SEM), whereas skewed variables are presented as median and interquartile range (IQR). All analyses were performed using two-sided statistical testing, and *p*-values < 0.05 were considered statistically significant. Graphs and figure visualizations were prepared using GraphPad Prism (version 7.0; GraphPad Software, San Diego, CA, USA).

#### Sample Size Determination/Power Analysis

Sample size determination was based on previously published experimental studies of methotrexate-induced hepatotoxicity using similar biochemical and histopathological endpoints and comparable group sizes [19,31,63]. In addition, sample size adequacy was evaluated using G*Power software (version 3.1.9.7) for one-way ANOVA with three groups. Power analysis was performed across major biochemical and histopathological endpoints, including plasma ALT, liver MDA, SIRT1, TGF-β, hydroxyproline, VEGF, SMAD3, hepatocyte necrosis, fibrosis, and cellular infiltration parameters. Based on the group means and pooled within-group standard deviations, the calculated effect sizes ranged from f = 0.72 to f = 2.39 with α = 0.05 and 80% statistical power. Therefore, including 10 rats per group (total *n* = 30) was considered sufficient to detect biologically meaningful differences among groups. No animals were excluded from the final analysis, and all animals survived until the planned termination of the experiment.

## 5. Limitations

Although the present model demonstrated clear profibrotic and collagen-associated changes following methotrexate exposure, the relatively short observation period and single-dose MTX protocol more likely reflect early fibrogenic remodeling rather than advanced established fibrosis. Therefore, the antifibrotic effects of Loureirin B should be interpreted within the context of acute-to-subacute fibrogenic liver injury. In addition, histopathological assessment was primarily based on H&E staining and semi-quantitative scoring; therefore, collagen-specific staining methods such as Masson’s trichrome or Sirius Red, together with α-SMA immunohistochemistry, would further strengthen the evaluation of fibrosis-associated alterations.

Furthermore, mechanistic causality could not be fully established because phosphorylated SMAD3, SIRT1 enzymatic activity, and downstream profibrotic targets were not assessed in the present study. In addition, the absence of a Loureirin B-only group limited the evaluation of the baseline hepatic effects of Loureirin B under physiological conditions. Furthermore, only female rats were included in the present study to maintain sex homogeneity within the experimental design; therefore, potential sex-dependent differences could not be evaluated.

Future studies incorporating collagen-specific staining, α-SMA expression, long-term fibrosis models, additional mechanistic analyses, liver weight assessment, and both sexes are warranted to validate these findings further.

## 6. Conclusions

In the present study, Loureirin B attenuated methotrexate-induced hepatic injury, as reflected by the reduced serum levels of liver enzymes, oxidative stress markers, TGF-β, hydroxyproline, and VEGF, along with partial histopathological improvement. These changes were accompanied by the partial restoration of hepatic SIRT1 levels and attenuation of fibrosis-associated alterations. Overall, the findings suggest that Loureirin B may exert hepatoprotective effects by modulating oxidative stress and profibrotic responses. However, because mechanistic pathway analyses were limited and the study used a short-term, single-dose MTX model, the findings should be interpreted in the context of early fibrogenic liver injury rather than advanced, established fibrosis.

## Figures and Tables

**Table 1:** Histopathological scores in liver tissue (median [IQR]).

Parameter	Normal Control (n = 10)	MTX + Vehicle (n = 10)	MTX + Loureirin B (n = 10)
Necrosis	0 [0–0]	1 [0.75–1] **	0 [0–1]
Fibrosis	0 [0–0]	2 [1–2.25] ***	1 [0–1] ##
Cellular infiltration	0 [0–0]	1 [1–2] ***	1 [0.75–1] **^,^#

Data are presented as median [interquartile range]. ** *p* < 0.01, *** *p* < 0.001 vs. normal control group; # *p* < 0.05, ## *p* < 0.01 vs. MTX group.

**Table 2:** Biochemical parameters in liver tissue.

Parameters	Normal Control (*n* = 10)	MTX + Vehicle (*n* = 10)	MTX + Loureirin B (*n* = 10)
Serum ALT (U/L)	39.63 ± 2.71	72.88 ± 3.75 ***	45.44 ± 2.40 ###
Serum AST (U/L)	88.50 ± 4.05	197.0 ± 11.89 ***	117.6 ± 5.24 *^,^###
Serum ALP (U/L)	174.0 ± 6.28	251.3 ± 8.64 ***	198.3 ± 6.20 ###
Liver MDA level (nmol/g tissue)	21.68 ± 1.29	40.23 ± 2.70 ***	29.74 ± 1.57 *^,^##
Liver SIRT-1 level (pg/mg)	14.99 ± 0.66	5.63 ± 0.43 ***	9.47 ± 0.88 ***^,^##
Liver TGF beta level (ng/g)	1.86 ± 0.15	4.18 ± 0.22 ***	2.21 ± 0.10 ###
Liver Hydroxyproline (ng/mg)	315.80 ± 18.61	1268.45 ± 80.85 ***	646.21 ± 42.55 ***^,^###
Liver SMAD3 level (pg/mg)	1.15 [0.93–1.30]	2.50 [2.21–3.66] ***	2.10 [1.86–2.43] ***
Liver VEGF (pg/mg)	118.90 ± 7.04	316.41 ± 22.21 ***	211.97 ± 14.08 ***^,^###

Data are presented as mean ± SEM for parametric variables and median [IQR] for SMAD3 (n = 10 per group). * *p* < 0.05, *** *p* < 0.001 vs. normal control group; ## *p* < 0.01, ### *p* < 0.001 vs. MTX group.

## Data Availability

The data presented in this study are available from the corresponding author upon reasonable request. The data are not publicly available due to ethical and privacy reasons.
